# Dietary restriction interventions: lifespan benefits need resilience and are limited by immune compromise and genetics

**DOI:** 10.1038/s41392-024-02074-x

**Published:** 2024-11-28

**Authors:** Yulin Chen, Anjalika Malik, Karl Lenhard Rudolph

**Affiliations:** Leibniz Institute on Aging – Fritz Lipmann Institute (FLI), Jena, Germany

**Keywords:** Molecular medicine, Experimental models of disease

A recent study published in *Nature*^1^ highlights how inter-individual differences in resilience can predict which individuals derive the most benefit from dietary restriction (DR) protocols—approaches shown to enhance health and longevity across species by mitigating age-related declines in cellular function.

DR involves limiting food intake or restricting eating windows. Key protocols include caloric restriction (CR), which reduces caloric intake without malnutrition, and time-restricted feeding (e.g., intermittent fasting, IF), which increases periods of starvation between meals. Both induce mild stress, activating nutrient-sensing pathways that promote cellular function. Resilience represents a broad concept from biomedicine and psychology, which refers to the capacity of organisms to maintain physiological stability under various types of stress. While resilience has been implicated in healthy aging, it may play a role in DR-induced, metabolic stress response and lifespan extension, though this remains uncertain. Di Francesco et al.^1^ used a genetically diverse mouse model to study the effects of 2 doses of CR (20% and 40%), and different durations of IF (1 or 2 days/week). The authors found that CR, especially at 40% restriction, extended lifespan 1.5 to 3 times more than IF. However, the more intense CR protocol reduced certain health markers, such as B-lymphocyte count and lean body mass, which could compromise disease resistance, especially in humans. This highlights the need for caution in applying CR or IF interventions for aging.

**Fig. 1:**
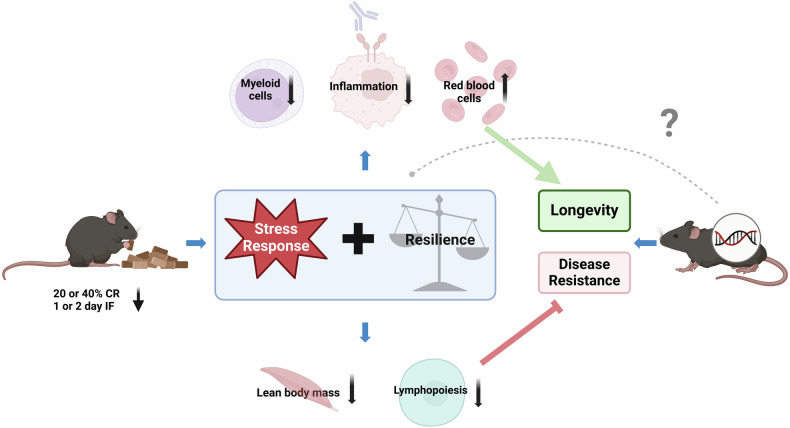
Dietary restriction needs resilience. Dietary restriction (DR) protocols, such as calorie restriction (CR) and intermittent fasting (IF), trigger stress responses that enhance longevity, particularly in individuals with high resilience who maintain body weight, adipose tissue, and blood lymphocyte levels. While CR has health-promoting effects—such as reducing myeloid cell production and inflammation while increasing red blood cell production—more intense protocols (40% restriction versus 20%) can have negative consequences, including declines in lymphopoiesis and lean body mass. The emergence of these adverse effects likely depends on the intrinsic resilience capacity of the individuum and could undermine the robustness and disease resistance of free-living organisms, including humans. The interplay between these positive and negative responses to nutrient restriction remains to be fully understood, particularly concerning organismal resilience. Notably, influences of inherited genetic factors on lifespan were found to be stronger than CR. It is conceivable that inherited genetic factors also influence the effectiveness of CR and/or resilience pathways in extending lifespan (grey, dotted line). Created with BioRender.com

The lowering of metabolism has been proposed as a key mechanism by which CR extends lifespan. Yet, Di Francesco et al.’s findings challenge this notion; reductions in mitochondrial respiration, blood glucose, and energy expenditure did not correlate with longevity. Instead, they identified resilience to aging and stress-induced weight loss, particularly the loss of white adipose tissues, as a key determinant of longevity across all dietary groups (Fig. 1). Although, CR promotes weight loss and adipose tissue reduction, resilience to these changes—evidenced by better maintenance of body weight and fat during stress—predicts greater longevity. It remains unclear whether stress- and age-induced losses of adiposity involve distinct pathways that govern aging and psychological stress, which may converge in regulation of body composition but have distinct and broad effects that likely affect longevity. Although Di Francesco et al.’s study does not identify the specific pathways by which resilience modulates body composition—pathways that may have far-reaching implications for longevity—it highlights the urgent need for further research to elucidate these mechanisms.

Interestingly, the activation of lipid storage and metabolism is also essential for the lifespan-extending effects of dietary supplementation of mono-unsaturated fatty acids in *Caenorhabditis elegans*.^2^ This suggests that resilience to stress-induced lipid loss may enhance the longevity effects of CR by maintaining the capacity of lipid metabolism. Further support for the concept of resilience comes from analyzing blood cell composition. Di Francesco et al. found that an individual’s resilience to age-related changes in blood cell makeup is a predictor of longevity (Fig. 1). Specifically, increases in myeloid cells and activated immune cells correlated negatively with lifespan, while the maintenance of naïve immune and red blood cells positively correlated. CR appeared to mitigate adverse age-related changes, suggesting it bolsters the resilience of the immune system.

Collectively, these insights suggest that resilience to nutrient stress may serve as a promising biomarker for identifying individuals poised to benefit most from CR and IF interventions. Nevertheless, the pathways that govern resilience, along with its organ-specific contributions to longevity, remain yet to be defined. It would be important to delineate how they interact with known homeostasis mechanisms governed by pathways that relate to aging such as DNA damage, inflammation, or nutrient signalling. This could also guide the development of more nuanced biomarkers of resilience that influence longevity in the context of DR protocols. Intriguingly, a meta-analysis encompassing various murine studies hints at the possibility that DR protocols may impair post-infection resilience, raising concerns about its potential compromise of immune function.^3^ In addition, DR protocols may also impair general fitness of the organism. It is known that DR compromises fertility in *C. elegans*, which could impact the population size and fitness of future generations.

Furthermore, the Di Francesco study highlights the significant role of genetic factors in lifespan, suggesting they may outweigh the effects of CR and IF. While genetics are generally thought to account for about 30% of interindividual aging variability, lifestyle and environmental factors contribute the remaining 70%. Given this, the modest impact of CR is striking, particularly since it is one of the most effective dietary interventions for aging. This raises important questions about additional factors influencing lifespan variation within a species that are not linked to genetics.

Research from the Stroustrup laboratory has demonstrated that lifespan heterogeneity within genetically uniform populations of *C. elegans* is significantly driven by inter-individual differences in the expression of genes that regulate physiological networks, resulting in stochastic variations in life expectancy.^4^ As interindividual heterogeneity in lifespan appeared not to be affected in the Di Francesco study, one might speculate that while DR protocols effectively delay aging, they do not change the variability in the expression of genes determining lifespan heterogeneity.

In terms of translation, the study by Di Francesco et al. highlights the need for new biomarkers to assess the therapeutic benefits of CR and IF, extending beyond metabolic effects to include markers of resilience. Caution is necessary when developing DR interventions, as disparities may emerge between lifespan extension and the preservation of organismal fitness or immune function. Given human exposure to pathogens, biomarkers that reflect immune health will be essential to avoid negative impacts on disease morbidity, particularly post-infection. Moreover, the challenge of developing effective DR strategies is compounded by their reduced efficacy when initiated later in life.^5^ Achieving compliance with such interventions starting in young adulthood may prove unrealistic, but public acceptance could shift dramatically if effective strategies are developed that can enhance health and lifespan in older populations.
